# The burden of visually diagnosed female genital schistosomiasis among women with infertility in the Volta Region of Ghana

**DOI:** 10.1186/s41182-024-00660-x

**Published:** 2025-02-25

**Authors:** Verner N. Orish, Gladys Kaba, Anthony K. Dah, Raymond S. Maalman, Micheal Amoh, Adu Appiah-Kubi, Wisdom Azanu, David Adzah, William R. Nyonator, Micheal B. Kumi, Diana S. Awutey-Hinidza, Irene Atachie, Portia Ahiaku, Precious K. Kwadzokpui, Adam A. Fatau, Cecila Smith-Togobo, Tai-Soon Yong, Young-soon Cho, Emmanuel S. K. Morhe, So Yoon Kim, Margaret Gyapong

**Affiliations:** 1 Department of Microbiology and Immunology, School of Medicine, University of Health and Allied Sciences, Ho, Volta Region Ghana; 2 Department of Biomedical Sciences, School of Basic and Biomedical Sciences, University of Health and Allied Sciences, Ho, Ghana; 3 Department of Obstetrics and Gynaecology, School of Medicine, University of Health and Allied Sciences, Ho, Ghana; 4 Department of Basic Medical Sciences, School of Medicine, University of Health and Allied Sciences, Ho, Ghana; 5 School of Medicine, University of Health and Allied Sciences, Ho, Ghana; 6 Ghana Health Service (Central Tongu District Health Directorate), Adidome, Ghana; 7 Department of Midwifery, School of Nursing and Midwifery, University of Health and Allied Sciences, Ho, Ghana; 8 Department of Obstetrics and Gynaecology, Ho Teaching Hospital, Ho, Volta Region Ghana; 9 Department of Medical Laboratory Sciences, School of Allied Sciences, University of Health and Allied Sciences, Ho, Ghana; 10 Laboratory Department, Ho Teaching Hospital, Ho, Volta Region Ghana; 11 College of Medicine, Yonsei University, Seoul, Republic of Korea; 12 Asian Institute for Ethics and Health Law, Graduate School of Public Health, Yonsei University, Seoul, Republic of Korea; 13 Asian Institute for Bioethics and Health Law, College of Medicine, Yonsei University, Seoul, Republic of Korea; 14 Institute of Health Research, University of Health and Allied Sciences, Ho, Volta Region Ghana

## Abstract

**Background:**

Female genital schistosomiasis (FGS) is the outcome of the deposition of *Schistosoma haematobium* egg in the ovaries, fallopian tubes, uterus or cervix of women in schistosomiasis endemic areas. Chronic and untreated FGS can result in an increased risk of human immunodeficiency virus (HIV) acquisition and infertility. This study aimed to evaluate the burden of visual FGS among women with infertility in the Volta region of Ghana.

**Methods:**

This study was a comparative cross-sectional study involving women with infertility defined as women with inability to achieve pregnancy after 12 months or more of frequent (3–4 times a week) unprotected sexual intercourse and nursing mothers (fertile women) from selected districts in the Volta Region. Questionnaire administration was used to obtain sociodemographic information including recent and childhood contact with water bodies as well as the practice of open defecation and clinical information such as the presence of genital symptoms. Urine samples were collected for detection of eggs of *S. haematobium*, and the women’s lower genital tracts were examined using a handheld colposcope by two gynecologists and a third to resolve discrepancies. Data were analyzed using SPSS version 23 with frequency distribution done for the sociodemographic variables and the prevalence of FGS in the women. Pearson Chi-square analysis was performed to find any significant difference between the prevalence of FGS among infertile and fertile women and any significant association between any socioeconomic and clinical variables with FGS. Logistics regression analysis was performed to investigate sociodemographic and other risk factors for FGS among women.

**Results:**

Of the 265 sampled women 132 (49.8%) were infertile and 133 (50.2%) were nursing mothers (fertile women). More women had visual FGS (155, 58.5%) and most with FGS were fertile [96, 76.1%; infertile, 59(45.3%); *p* < 0.001], with infertile women having lower odds of FGS in this study (AOR, 0.29 [95% CI 0.17–0.50]; *p* < 0.001); adjusted for childhood and current contact with rivers and streams, availability of toilets facility, practice of open defecation and age. More women with FGS had childhood contact with rivers and streams (68.4%, *p* = 0.007) with lower odds of FGS seen in women without childhood contact with rivers and streams (AOR, 0.52 [95% CI 0.31–0.88]; *p* = 0.015).

**Conclusion:**

In this study, infertile women unexpectedly had lower odds of FGS suggesting the need for more rigorous research on this topic to elucidate the true contribution of FGS on infertility.

## Introduction

Urogenital schistosomiasis is associated with clinical manifestations in the bladder and genital tract by the presence of *Schistosoma haematobium* eggs which can result in egg-induced lesions in the urinary bladder and genital tract [1–3]. Chronic deposited schistosome eggs within regions of the female genital tract such as the vagina, cervix, vulva, uterus, ovaries and fallopian tubes, can lead to a condition known as female genital schistosomiasis (FGS) [1–3]. Unlike FGS, urinary schistosomiasis is characterized by either painless or painful terminal hematuria, and with the presence of S. *haematobium* eggs in the urine, it is an easily recognizable tropical disease, common in sub-Saharan Africa [4, 5]. Despite its significance, FGS receives less attention and awareness than urinary schistosomiasis among healthcare providers and communities in endemic regions [4–6]. Currently, FGS is diagnosed according to the WHO FGS Pocket Atlas by cervix/vagina wall visualization during pelvic examination (aided by colposcopy), for characteristic lesions due to the chronic presence of Schistosoma eggs; such as grainy sandy patches, homogenous yellow sandy patches, rubbery papules, and abnormal blood vessels [7]. This WHO-backed visual diagnosis of FGS is a conventional diagnostic method but not a gold standard for diagnosis [7]. *S. haematobium* is the most implicated species to cause FGS probably because it is the most predominant species in endemic areas and the anatomical proximity of the adult worm (vesical and utero-vaginal plexus) to the genital tract [8, 9]. Overall, *S. haematobium* is reported to be responsible for about 230 million cases of both urinary schistosomiasis and FGS distributed in 54 countries worldwide [3, 10]. It is estimated that between 20 to 56 million girls and women are infected with FGS in sub-Saharan Africa, a conservative estimation since most cases of FGS go undiagnosed [4, 11, 12]. The egg-induced inflammatory response in the genital tract results in symptoms such as vaginal discharge and itching, vaginal bleeding or spotting after sexual intercourse, prepubertal bleeding and dyspareunia (painful sexual intercourse). If left untreated, FGS can lead to serious complications, including increased risk of human immunodeficiency virus (HIV) infection, ectopic pregnancy and infertility [13, 14]. The infertility in FGS is due to *Schistosoma haematobium* eggs-induced inflammatory lesions in the genital tract such as chronic cervicitis, granulomatous endometritis, and blockage of uterine tubes [15]. Case report studies as well as spatial distribution exploration studies have reported links and associations between FGS and infertility [16, 17].

Infertility is defined as when a woman who has never gotten pregnant (primary) or a woman who has been pregnant previously (secondary) fails to achieve conception or pregnancy after 12 months or more of regular unprotected sexual intercourse [18]. Reports have varied prevalence worldwide, but it is a consensus that the risk of infertility is highest in sub-Saharan Africa [19–23]. Diseases of the upper and lower genital tract have been implicated as the cause of infertility among women and tubal pathologies have been found to be the most common cause among women in sub-Saharan Africa [18, 24, 25]. Ultrasound and radiological techniques such as hysterosalpingography have been commonly used to detect tubal pathologies in women with infertility in sub-Saharan with few undergoing further microbiological investigation confirming the underlying diseases, such as Chlamydia, Gonorrhea, and tubal tuberculosis as the cause of the infertility [26–30]. With studies reporting associations between FGS and infertility coupled with prevalence studies reporting high prevalence of infertility in coastal and riparian areas in Africa further studies must be carried out [17, 30, 31].

In Ghana, there is a double whammy of infertility and schistosomiasis endemicity with the prevalence of infertility ranging between 2 and 14% [32] and a reported prevalence of urinary schistosomiasis between 50 and 60% in some parts of Ghana [33]. However, there is paucity of information regarding the prevalence of FGS in Ghana. The only report in 2011 reported a prevalence of 10.7% of FGS in the lower Volta Basin of the Volta region [34]. More so, there are no reports on the contribution of FGS to infertility or the association between infertility and FGS. This study, therefore, aimed to evaluate the burden of FGS among women with infertility in the Volta region.

## Methodology

### Study design and site

**Fig. 1:**
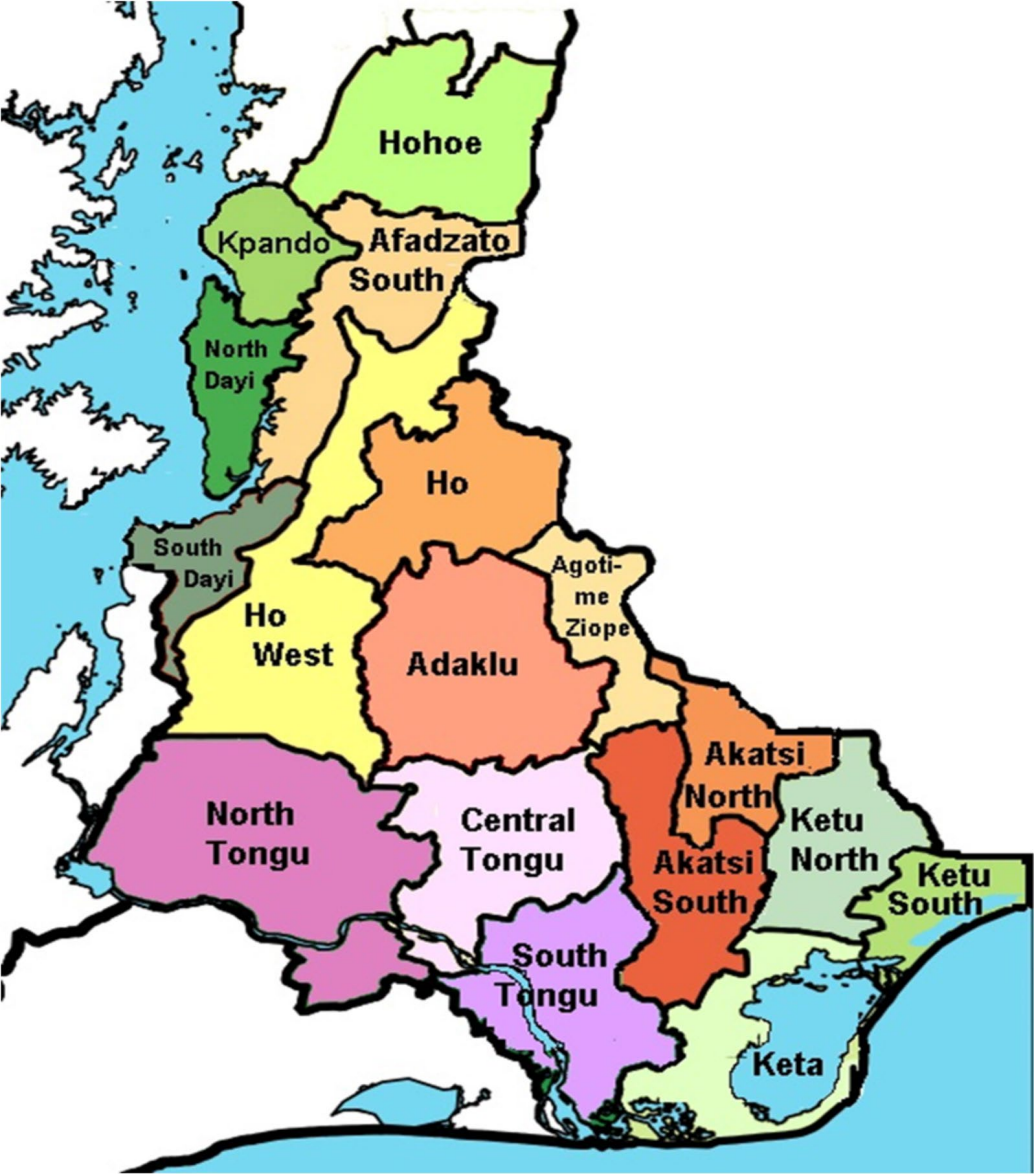
Showing the districts in southern Volta with the Volta Lake in blue

This was a comparative cross-sectional study involving a one-time encounter with infertile women of reproductive age and nursing mothers (fertile women), conducted in the Volta region of Ghana. The region is one of the 16 administrative regions in Ghana [35]. The Volta region is located between latitudes 50 45 N and 80 45ʺ N and located along the southern half of the eastern border of Ghana. It shares the border with the Republic of Togo. The region shares boundaries to the west with the Greater Accra, Eastern, Ahafo, and Oti regions, and has the Gulf of Guinea to the south. Volta Lake runs through the region from the south to the north, traversing several districts and towns (Fig. 1). Towns and villages around the lake and other rivers are historically known for a high prevalence of urinary schistosomiasis with close to 50% prevalence among school children [34, 36].

### Study population and selection procedure

This study involved women of reproductive age (15–49 years) attending hospitals or clinics for infertility treatment and nursing mothers attending hospitals or clinics for post-natal review or child welfare clinics. Women from Central Tongu, South Tongu, Akatsi, Ho Municipal, South Dayi and other districts were selected for this study. The sampling frame of this study for women with infertility was the register of the infertility clinic in health facilities of the selected districts. Only women who have been diagnosed using the standard diagnostic criteria for infertility [18], were included in this study, those with established anovulatory problems secondary to hormonal imbalance as well as women whose spouses are also being treated for infertility were excluded from the study. A health promotion officer was selected to help contact these women through phone calls or home visits in the case of the absence of phone contacts.

Fertile women were recruited from child welfare and post-natal clinics after providing informed consent. They included healthy nursing mothers who were between 6 weeks and 12 months postpartum. A convenient sampling method was employed to select the participants for this study. These women were all permanent residents of the selected districts or had stayed in the districts for a minimum of 12 months.

### Sample size determination

Using the Cochran formula for sample size calculation with a confidence interval of 95%, a margin of error was set as 5%, and using a 10.6% prevalence of FGS among women of reproductive age in the Volta region [34], a minimum sample size of 145 was obtained. The sample size was increased to 265 participants due to the comparative cross-sectional design of the study, which included two district groups. To ensure sufficient statistical power for subgroup analysis, it was necessary to recruit a larger sample to achieve significant results within each group. This approach allows for more reliable comparisons and enhances the robustness of our findings accross both study populations.

### Data collection procedure

The women were invited to the health facilities in their districts for the study, which took place between 9.30 a.m. to 3:00 p.m. each day of the data collection. After consent was obtained, the women were made comfortable and data collection commenced. Data collection took place from July to November 2023.

### Validation of data collection instruments and questionnaire administration

Prior to the administration of the structured close-ended questionnaire, which was created anew for this study, a thorough face validation exercise was done with two independent experts. They objectively reviewed and assessed the questionnaire, ensuring that it was fit for purpose, the questions were appropriate, and the language was simple, clear and comprehensible before the commencement of data collection.

The questionnaire was used to obtain the sociodemographic characteristics of the women, contact with water bodies in childhood and current contact, frequency, duration, and nature of contact, and others were obtained. Clinical information such as the history of blood in urine, vaginal discharge, and vaginal bleeding, among others was also obtained. The history of infertility (primary and secondary), miscarriages, and ectopic pregnancy were also obtained.

### Urine sample collection and *Schistosoma* egg detection

Fresh urine samples were collected from the women on the morning of the data collection in the health facility. Urine collection was done with a sterile container and transported to the laboratory for analysis on the same day. The samples were subjected to the sedimentation method using centrifugation without filtration. The sediment was covered with a clean 24 × 24 mm cover glass in preparation for microscopic examination. The *entire* sediment was examined microscopically for *S. haematobium* eggs or miracidia using the 10X objective lens with the condenser iris closed sufficiently to give a good contrast.

### Pelvic examination and diagnoses of FGS

Pelvic examination was performed using a handheld colposcope (EVA Systems—Mobile ODT, Tel Aviv, Israel) by trained personnel using a disposable plastic speculum. The images obtained were sent electronically to the team of expert consultant gynecologists who were blinded to the clinical and demographic characteristics of the women. Colposcopy was done for both fertile and infertile women to identify FGS lesions induced by *Schistosoma haematobium* eggs in the vaginal wall and cervix such as grainy sandy patches, homogeneous yellow sandy patches, rubbery papules, and abnormal blood vessels. If there are any of these lesions noted, a diagnosis of FGS was made, and it was termed “visual FGS”. This was done after two gynecologists reviewed the images and agreed to the presence and type of the FGS lesion after comparing them with the WHO FGS atlas. If there was a disagreement between the two gynecologists a third gynecologist reviewed and gave a final verdict. Women with suspected FGS lesions or any other abnormal lesions were counseled on the findings and referred for management. All the women were educated on the problems and prevention FGS.

### Statistical analysis

Data collected were analyzed using the Statistical Package for Social Science (SPSS) version 23. Frequency distribution was done for the sociodemographic variables in the study and the prevalence of FGS in the women of the study. A Chi-square analysis was performed to find any significant difference between the prevalence of FGS among women with infertility and those without infertility and any significant association between any socioeconomic variables and FGS. Chi-square was also used to find out the association between clinical variables and FGS. Logistics regression analysis was performed to investigate sociodemographic and other risk factors for FGS among women. All analysis was performed using 95% confidence intervals and the statistical significance was set at *p* < 0.05.

## Result

### Socio-demographic characteristics of the participants

A total of 265 women were recruited for this study and the majority are from Central Tongu district (124, 46.6%), followed by Ho municipal (49, 17.3%), Akatsi (41, 15.4%), South Tongu (31, 11.7%) and South Danyi (15, 5.6%). Out of 265 participants, majority of the participants were married (194, 72.9%), had junior high school education (JHS) (101, 38%), were Christians (254, 95.8%), 70 participants were traders (26.3%), 167 (63%) had their source of drinking water from tap water and majority had contact with water bodies in childhood (136, 51.1%), no current contact with water bodies (159, 59.8%). Domestic chores (85, 80.1%) were the most common type of activity resulting in contact with water bodies and most contacts occurred daily (44, 16.5%). A total of 71(26.8%) participants had no toilet in their household and 62 (23.4%) participants practiced open defecation as presented in Table 1.

**Table 1:** Socio-demographic characteristics of the participants

Characteristics	Frequency	%
Districts		
Afadzato-south district	1	0.4
Agotime-Ziope	1	0.4
Akatsi	41	15.4
Centra Tongu	124	46.6
Ho	49	17.3
Hohoe	1	0.4
Ketu South	1	0.4
Kpando	1	0.4
South Danyi	15	5.6
South Tongu	31	11.7
Age		
Mean age ± SD	32.7 ± 7.42	
15–19	8	3.0
20–35	155	58.5
36–49	102	34.5
Education status		
No formal education	12	4.5
Primary	48	18
JHS	101	38
SHS	41	15.4
Tertiary	60	22.6
Postgraduate	3	1.1
Religion		
Christianity	254	95.8
Islam	4	1.5
Traditional	6	2.3
Atheist	1	0.4
Occupation		
Farming	54	20.3
Trading	70	26.3
Artisan	46	17.3
Civil servants	57	21.4
Unemployed	38	14.3
Marital status		
Single	25	9.4
Married	194	72.9
Divorced/separated	4	1.5
Cohabiting	42	15.8
Source of drinking water		
River	3	1.1
Rainwater	1	0.3
Well water	2	0.7
Borehole	1	0.3
Tap	167	63
Mineral water	91	34.3
Childhood contact with river/stream		
Contact	136	51.3
No contact	129	48.7
Current contact with river/stream		
Contact	106	39.8
No contact	159	59.8
Type of contact		
Swimming	2	1.8
Domestic chores	85	80.2
Fishing	2	1.8
Passing through water	3	3.8
All	14	13.2
Frequency of contact		
Daily	44	41.5
2–3/weekly	34	32.1
Once a week	12	11.3
Once a month	10	9.4
Once a year	6	5.6
Presence of household toilet		
No	71	26.8
Yes	194	73.2
Type of toilet		
Open defecation	62	23.4
Traditional	114	43.0
Modern	89	35.8

### Clinical characteristics of the participants and type of lesions seen among with visual FGS

This study recruited 132 (49.8%) women with infertility and 133 (50.2%) nursing mothers (fertile women). A total of 58.5% of women had visual FGS and 38.1% had normal colposcopy findings. Of the overall 265 study participants, only 5(1.9%) and 92(34.6%) of the participants affirmed to have had ectopic pregnancy and miscarriage, respectively. The women reported a variety of genitourinary symptoms with hematuria (5.6%) being the least of the symptoms reported while vaginal itching (38.1%) followed by vaginal discharge (35.1%) was common. In all, 173 (65.3%) participants reported at least one genitourinary symptom while 34.7% reported no symptom at all as shown in Table 2.

**Table 2:** Clinical characteristics of the participants

Characteristics	Frequency	%
Fertility status		
Infertile	132	49.8
Fertile	133	50.2
Type of infertility		
Primary	64	48.5
Secondary	68	51.5
Urine Schistosoma egg detection		
Yes	0	0.0
No	265	100.0
Colposcopy		
Normal	101	38.1
Visual FGS	155	58..5
Undetermined	9	3.4
History of reproductive system conditions		
Ectopic	5	1.9
Miscarriage	92	34.6
Sexually transmitted infection	34	12.8
Symptoms affecting the genital tract		
Vaginal discharge	93	35.1
Bloody vaginal discharge	19	7.2
Dyspareunia	72	27.2
Vaginal itching	101	38.1
Vaginal lumps swelling	25	9.4
Symptoms affecting the urinary tract		
Hematuria	15	5.6
Dysuria	62	23.4
Urinary incontinence	24	9.1

^*^NB—167/265 (63.0%) of study participants reported at least one genital symptom

**Fig. 2:**
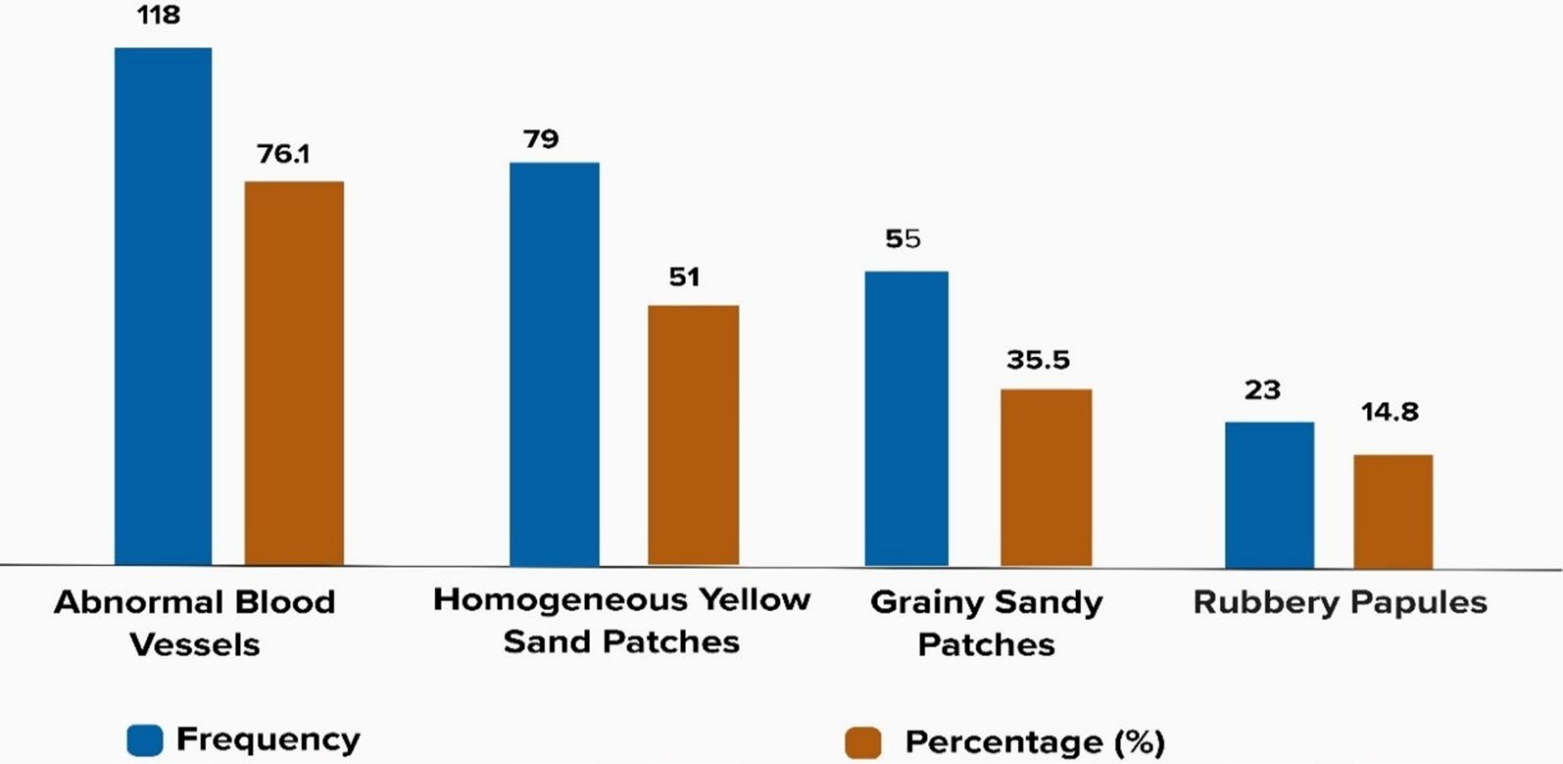
Types of lesions seen in the women with visual FGS

**Fig. 3:**
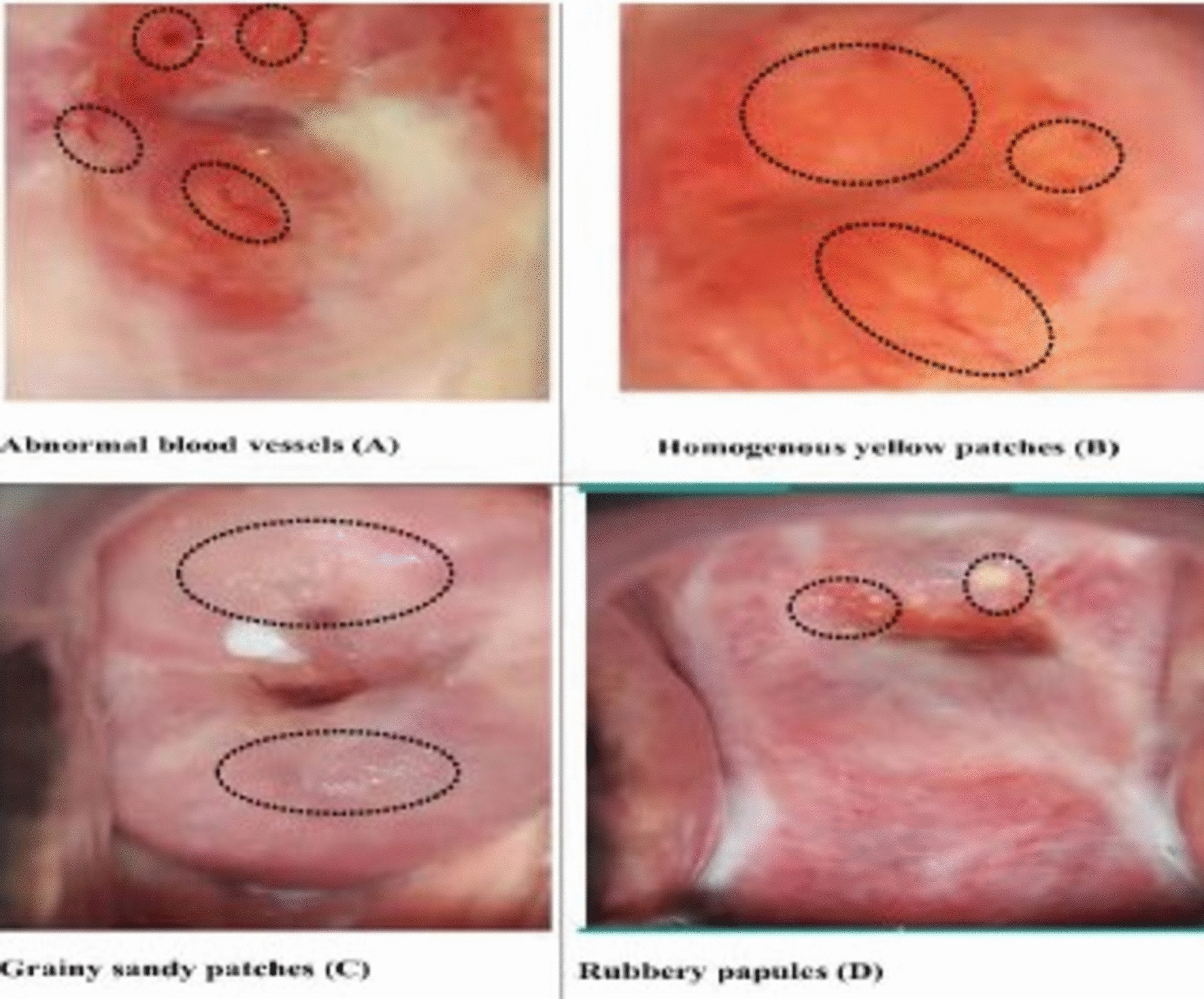
Images of FGS lesions

Figure 2 shows that the majority of the characteristic FGS lesions seen among women with visual FGS were abnormal blood vessels (Fig. 3A) (118,76.1%), homogenous yellow sandy patches (Fig. 3B) (79, 62.6%), grainy sandy (Fig. 3C) (55 35.5%) and the least lesions seen was rubbery papules (Fig. 3D) (23, 14.8.5%).

### Socio-demographic and clinical characteristics of women stratified by colposcopy findings

Table 3 shows the sociodemographic characteristics of the women stratified by colposcopy findings-visual FGS and normal. For districts, the women from Akatsi (84.2%), South Tongu (66.6%), and Central Tongu (65.5%) significantly had more visual FGS (*p* < 0.001). Proportionally, women with childhood contact with rivers and streams (68.4%) significantly had more FGS compared with those who had no contact (52.0%) (*p* = 0.001). Similar findings were noted among women with current contact with streams and rivers (69.6%) (*p* = 0.016). Women without toilets in their household (74.6%, *p* = 0.007) and those who practice open defecation (77.2%, *p* = 0.013) proportionally had more visual FGS.

**Table 3:** Socio-demographic characteristics of women stratified by colposcopy findings

Characteristic	Normal (%)	Visual FGS (%)	Total	*p* value
Districts				<0.001
Afadzato South District	1(100)	0(0)	1	
Agotime-Ziope	1(100)	0(0)	1	
Akatsi	6(15.8)	32(84.2)	38	
Central Tongu	41(33.9)	78(65.5)	119	
South Tongu	10(33.3)	20(66.7)	30	
Ho	32(65.3)	17(34.7)	49	
Hohoe	1(100)	0(0)	1	
Ketu South	1(100)	0(0)	1	
Kpando	0(0)	1(100)	1	
South Danyi	8(53.3)	7(46.6)	15	
Age				
15–19	3(37.5)	5(62.5)	8	0.044
20–35	50(32.9)	100(67.1)	149	
36–49	48(49.0)	50(51.0)	98	
Childhood contact with river/stream				
No	59(48.0)	64(52.0)	123	0.007
Yes	42(31.6)	91(68.4)	133	
Current contact with river/stream				
No	70(45.5)	84(54.5)	154	0.016
Yes	31(30.4)	71(69.6)	102	
Frequency of contact				
Daily	16(38.1)	26(61.9)	42	0.070
2–3 times weekly	13(40.6)	19(59.4)	32	
One a week	1(8.3)	11(91.7)	12	
Once a month	1(10)	9(90)	10	
Once a year	0(0)	6(100)	3	
Source of drinking water				
River	1(50)	1(50)	2	0.621
Well water	0(0)	2(100)	2	
Borehole	0(0)	1(100)	1	
Tap	67(41.4)	95(56.6)	162	
Mineral water	32(36.4)	56(63.6)	88	
Marital status				
Married	77(41.4)	109(58.6)	186	0.113
Cohabiting	8(19.5)	33(80.5)	41	
Single	14(58.3)	10(41.7)	24	
Divorced/separated	1(25)	3(75)	4	
Education				
No formal education	5(45.5)	6(54.5)	11	0.115
Primary	13(27.7)	34(72.3)	47	
JHS	34(35.1)	63(64.9)	97	
SHS	16(40)	24(60)	40	
Tertiary	30(52.6)	27(47.4)	57	
Postgraduate	2(66.7)	1(33.3)	3	
Occupation				
Unemployed	13(38.2)	21(61.7)	34	0.080
Trader	19(28.4)	48(7.6)	67	
Farmer	20(38.5)	32(61.5)	52	
Civil servant	30(53.6)	26(46.4)	56	
Artisan	18(39.1)	28(60.8)	46	
Religion				
Christian	99(40.2)	147(59.8)	246	0.312
Islam	0(0)	2(100)	2	
Traditional	1(16.7)	5(83.3)	6	
Atheist	0(0)	1(100)	1	
Presence of household toilet				
No	17(25.4)	50(74.6)	67	0.007
Yes	83(44.1)	105(55.9)	188	
Type of toilet				
Modern	48(43.6)	62(56.4)	110	0.013
Traditional	40(44.5)	48(55.5)	88	
Open defecation	13(22.8)	44(77.2)	57	

Table 4 highlights the clinical characteristics of the participants stratified by colposcopy findings. Fertile women (96, 76.1%) significantly had more visual FGS compared to the women with infertility (59, 45.3%) (*p* < 0.001). Women who reported at least one genital symptom (106, 65.4%) had more visual FGS compared to those who had no genital symptoms (49, 52.1%) (*p* = 0.04).

**Table 4:** Clinical characteristics of women stratified by colposcopy findings

Characteristics	Normal (%)	Visual FGS (%)	Total	*p* value
Fertility status				
Infertile	71(54.6)	59(45.3)	130	<0.001
Fertile	30(23.8)	96(76.1)	126	
Miscarriage				
No	70(42.2)	96(57.8)	166	0.223
Yes	30(33.7)	59(66.3)	89	
Ectopic pregnancy				
No	99(39.4)	152(60.6)	251	0.981
Yes	2(40)	3(60)	5	
History of previous STI				
No	85(38.2)	137(61.7)	222	0.324
Yes	16(47.1)	18(52.9)	34	
Vaginal discharge				
No	74(44.8)	91(55.2)	165	0.017
Yes	27(29.6)	64(70.3)	91	
Bloody vaginal discharge				
No	95(39.9)	143(60.1)	238	0.400
Yes	6(33.3)	12(66.7)	18	
Dyspareunia				
No	75(40.5)	110(59.5)	185	0.610
Yes	24(34.8)	45(65.2)	69	
Vaginal itching				
No	67(42.1)	92(57.9)	159	0.312
Yes	34(35.1)	63(64.9)	97	
Reported at least one genital symptom				
No	45(47.9)	49(52.1)	94	0.042
Yes	56(34.6)	106(65.4)	162	

*STI* sexually transmitted infection

### Multiple logistic regression for the odds of FGS among participants

Table 5 shows the multivariate logistic analysis for the odds of visual FGS among the participants. Infertile women significantly had lower odds of having visual FGS (unadjusted OR 0.26 [95% CI 0.15–0.44]; *p* < 0.001) and significantly lower odds remained (adjusted OR 0.29 [95% CI 0.17–0.50, *p* < 0.001]) after adjusting for history of childhood and current contact with river or streams, availability of toilet facility at home, the practice of open defecation and age. Women who had no history of childhood water contact had lower odds of having visual FGS (unadjusted OR 0.50, [95% CI 0.30–0.83], *p* = 0.008) and after adjusting for the categories of participants, history of current contact with river or streams, availability of toilet facility at home and the practice of open defecation, significant lower odds remained (adjusted OR 0.52, [95%CI 0.31–0.88]; *p* = 0.015). Similarly, those without current contact with river or stream had lower odd/risk (unadjusted OR 0.52 [95% CI 0.31–0.88], *p* = 0.02) however in contrast, the significance disappeared after adjusting for the other factors (adjusted OR 0.54 CI 0.38–0.91), *p* = 0.06). Women without toilet facilities within their homes (unadjusted OR 2.30 CI 1.25–4.33, *p* = 0.005) and those that practiced open defecation (unadjusted OR 2.89, CI 0.37–6.08, *p* = 0.008) had twice the risk/odds of having visual FGS, however after adjusting for the other factors, the significance was lost for women without toilet (adjusted OR 2.19, CI 1.17–4.12. *p* = 0.074) and women who practiced open defecation (adjusted OR 2.68, CI 1.26–5.67, *p* = 0.08). Women without symptoms of vaginal discharge had lower odds of having visual FGS (unadjusted OR 0.52, [95% CI 0.30–0.89], *p* = 0.05) as well as women who reported no genital symptoms (unadjusted OR 0.58, [95% CI 0.34–0.97], *p* = 0.04). After adjusting for the categories of participants, history of childhood and current contact with rivers or streams, availability of toilet facility at home and the practice of open defecation the significance remained for both women with no symptom of vaginal discharge (adjusted OR = 0.60, [95% CI 0.34–1.05], *p* = 0.08) and those who did not report any urogenital symptoms (adjusted OR = 0.65, [95% CI 0.38–1.10], *p* = 0.11).

**Table 5:** Logistic regression for the odds of visual FGS among participants

Characteristics	Unadjusted OR (95% CI)	* p value*	Adjusted OR (95% CI)	* p value*
Fertility status				
Infertile	0.26 (0.15–0.44)	< 0.001	0.29 (0.17–0.50)	<0.001
Fertile	1		1	
Age				
15–19	0.63 (0.14–2.76)	0.520	0.66(0.14–3.13)	0.550
20–35	0.51 (0.30–0.86)	0.120	0.50(0.29–0.87)	0.130
36–49	1		1	
Childhood contact with river/stream				
No	0.50 (0.30–0.83)	0.008	0.52 (0.31–0.88)	0.015
Yes	1		1	
Current contact with river/stream				
No	0.52 (0.31–0.89)	0.020	0.54 (0.31–0.96)	0.062
Yes	1		1	
Toilet facility at home				
No	2.30 (1.25–4.33)	0.008	2.19 (1.17–4.12)	0.074
Yes	1		1	
Type of toilet facility				
Open defecation	2.89 (1.37–6.08)	0.005	2..68 (1.26–5.67)	0.081
Traditional	1.08 (0.61–1.89)	0.860	1.16 (0.65–2.05)	0.623
Modern	1		1	
Vaginal discharge				
No	0.52 (0.30–0.89)	0.018	0.60 (0.34–1.05)	0.043
Yes	1		1	
Reported at least one genital symptom				
No	0.58 (0.34–0.97)	0.040	0.65 (0.38–1.10)	0.051
Yes	1		1	

## Discussion

### Diagnosis of FGS

The diagnosis of FGS in this study was made using the visual FGS technique from images obtained with the handheld colposcope. This is a widely used clinical diagnosis employed by several studies [11, 37], after approval by a team of experts in a consensus meeting held in Denmark in 2010 [8]. The WHO further endorsed this consensus with the publication of the WHO FGS pocket atlas, a reference tool to be used by health professionals in schistosomiasis endemic areas to aid in the clinical diagnosis of FGS [7]. Inherent to most clinical diagnostic methods, visual FGS lacks specificity as the lesions noted for FGS have also been reported in the early phase of cervical cancer, herpes simplex virus and human papillomavirus infections [11, 37, 38]. Restricted anatomical limitations is another weakness as the colposcope is unable to view beyond the lower genital tract of the vaginal wall and the cervix, thus missing the upper genital tract, another common site for egg deposition and FGS lesions [8, 13, 39]. Lastly, the human error (inter-observer error) aspect of the diagnosis is another challenge as interpreting the images despite the use of WHO FGS atlas has some subjectivity resulting in contrasting results among expert reviewers [37, 38]. Despite these limitations with visual FGS, it remains the most feasible and practical diagnostic method in resource limited Schistosoma-endemic areas compared to the expensive molecular technique and the increased risk of HIV infection from the biopsy method [8, 13, 40]. Molecular testing of home-based self-collected sampling could have been a more feasible option except for the lack of expertise and logistics in resource limited endemic areas coupled with as the expensive molecular diagnosis [8]. However visual FGS diagnosis has contributed greatly to elucidating the burden of FGS in schistosomiasis endemic areas [2, 41].

### Prevalence of FGS

Our study reported a 58.5% prevalence of visual FGS among the study participants a rather high prevalence found as compared to a prevalence of 10.6% in the only prevalence study so far in literature done in the same region of Ghana as our study [34]. While our study and that of Yirenya-Tawiah et al. [34] share similar epidemiological characteristics (study site and demography of the participating women), their diagnostic method was punch biopsy which was collected from only women with suspected vaginal or cervical lesions ascertained by direct speculum inspection of the vagina and cervix without the aid of a colposcope which might explain the low prevalence obtained in their study. However, visual FGS is less sensitive compared to biopsy method [8]. Some other studies in Nigeria have also reported a lower prevalence of visual FGS such as 7.5% in Anambra state [42], and 15.4% in Ogun state [14]. These studies despite using colposcopy in the diagnosis of FGS, probably reported a lower prevalence due to the method of selecting only women with confirmed urinary schistosomiasis for colposcopy [42] or using a smaller sample size [14] However, some studies in Africa have reported a high prevalence of visual FGS such as 50.6% reported in Cameroun [43], 35.3% and 63.6% in Zambia [37], 26.9% in Malawi [11]. Taken together, all these studies share similar socioeconomic, epidemiologic and environmental similarities with our study, however variations in methods might be responsible for the differences in the reported FGS prevalence.

### Epidemiological risk factors

The high prevalence of FGS in this study might also be due to the historical precedence of schistosomiasis endemicity in the Volta region because of the Volta Lake which was created in 1964 from the Volta River as part of electricity and other water projects [44]. The participants in this study where particular at risk because part of the Volta Lake and other rivers runs through their communities and villages, increasing their risk of contact with these water bodies [35]. Our results buttress this, as the majority of these women (51%) had childhood contact with rivers or streams and some (40%) still had contact as adults, with domestic chores being the prominent reason for these contacts, which is a common finding in endemic areas [45, 46]. Visual FGS was significantly associated with childhood water contact in this study (*p* = 0.007), as more women with childhood contact with river and streams had more visual FGS (68.8%) than women without childhood contact (52%) who also showed lower odds of visual FGS (Adjusted OR 0.52[95% CI 0.31–0.88]; *p* = 0.015).

It has been widely reported that FGS is often acquired from childhood [40, 47, 48]. This is probably due to the behavioral tendencies of children to frequently visit rivers and streams not only for domestic chores, but also for recreational purposes like swimming, bathing and others, increasing the risk of infection [42, 49]. Our study also found significant association with and higher odds of visual FGS among women who practice open defecation or lack toilet facilities in their homes. This finding suggests the likelihood of pervading unsanitary practice of open defecation in the communities of the women, a practice that has been implicated as the source of contamination of water bodies aiding transmission of schistosomiasis [50–52].

### Prevalence of urinary schistosomiasis

Although 15/265 (5.6%) of our study participants presented with hematuria, *Schistosoma haematobium* eggs were not detected via light microscopic viewing of urine sediments among the participants in this study, a situation that is not entirely surprising because FGS can occur without urinary schistosomiasis [47, 53, 54]. However, it is apropos to also consider the impact of the method used in the detection of eggs of haematobium would have had on the absence of urinary schistosomiasis. The Sedimentation method using centrifugation without filtration employed in this study might have contributed to absence of urinary schistosomiasis as filtration technique would have concentrated any eggs in the urine sediment increasing the chance of detection in microscopy [55]. More so, the single urine collection from the participants might have hampered the diagnosis of urinary schistosomiasis because the sensitivity of the microscopic method improves with multiple sample collections [56–58].

### Prevalence of visual FGS among women with infertility

One of the major goals of this study was to find out the burden of visual FGS among women with infertility and to the best of our knowledge this is the first study of this nature in Ghana. Of the 130 infertile women who had a pelvic examination with colposcopy, about 45% had visual FGS, lower than 76% of the 126 fertile women (nursing mothers). Our study further shows that infertile women had lower odds of visual FGS (Adjusted OR 0.29 [95% 0.17–0.50]; *p* < 0.001). This was an unexpected finding as reports have suggested that FGS is linked with infertility among women living in schistosomiasis endemic areas [15–17]. Despite the postulations of possible hormonal disturbance and local immune response disruptions of the genital tract induced by *Schistosoma haematobium* eggs as the cause of infertility [30, 59], arguments and reports supporting mechanical disruptions of tubal motility secondary to granuloma and fibrosis formation as the major cause of infertility in FGS [15, 60, 61]. Thus, FGS lesions of the upper genital tract, which is unfortunately not visualized by colposcopy might contribute more to infertility than FGS of the lower genital tract. This might explain why visual FGS (lower genital tract lesions) was seen more among fertile women (nursing mothers) than the infertile women in our study, who might have had more of the FGS lesions in their upper genital tracts which is beyond the view of the colposcopy.

### Type of FGS lesions

The type of lesions seen among the participants with visual FGS in our study is very consistent with other findings of studies and reports [11, 37]. These lesions include abnormal blood vessels (76.1%), homogenous yellow sandy patches (51%), grainy sandy patches (35.5%) and rubbery papules (14.8%). The most prominent lesion in our study is the abnormal blood vessels, a very easy lesion to identify and usually accompanies the sandy patches (homogenous or grainy) [47, 62]. Grainy sandy patches usually suggest the presence of Schistosoma worm in the genital plexus and homogenous yellow sandy patches is suggestive of chronic egg deposition [63]. The least lesion identified among the women with visual FGS in this study was rubbery papules, a finding consistent with other reports as it is known to be found in much younger women [13, 64]. Rubbery papules suggest a recent egg deposition evidenced by active localized inflammatory infiltration of innate immune cells [64].

### Clinical manifestation of FGS

Infertility is not the only complication of FGS, there are others such ectopic pregnancy, miscarriages and varied urogenital clinical manifestations [8, 13, 16]. Ectopic pregnancy and miscarriage were reported among some women in this study but there was no significant association with visual FGS in this study. Some of the women in this study complained of varied genital symptoms such as vaginal discharge, vaginal itching, bloody vaginal discharge, and others, however taken together, more women with visual FGS significantly complained of at least one genital symptom (65%) and those who did not complain were less likely to have visual FGS (Adjusted OR 0.65[95% CI 0.38–1.10], *p* = 0.05). Similarly, vaginal discharge (70%) was proportionally seen more in women with FGS and those without vaginal discharge were less likely to be with visual FGS (adjusted OR 0.60 CI [95% CI 0.34–1.05]; *p* = 0.04). These findings are in keeping with several studies that have reported several nonspecific genital symptoms among FGS cases causing inaccurate diagnoses of sexually transmitted infection (STIs) or other genital disorders among these cases [13, 14, 34, 64, 65]. Additionally, Schistosoma eggs induce lesions in the lower female genital region that can lead to thinning, erosion, and ulceration of the epithelium leading to higher susceptibility to STIs including HIV and human papilloma virus (HPV) have been suggested to be a likely and/or associated with FGS cases with increased risk of AIDS and cervical cancer [2, 7, 66]. Thus, according to the WHO, FGS screening/prevention strategies when coupled with cervical cancer and HIV/AIDS control programs can be very important for reducing the three disease burden, especially in Africa [1].

### Limitations

Some limitations have previously been mentioned, however there is a unique limitation of this study concerning the infertile women that should be mentioned at this point. Even though women with infertility were accurately diagnosed with the standard criteria for the diagnoses of infertility by the qualified specialists in the respective health facilities we recruited them from, we did not discriminate further and confirm if their inability to conceive is not a problem of their male partners or spouse. Although the current conventional standard for FGS diagnosis was applied in this study, further laboratory investigation like molecular profiling of Schistosoma deoxyribonucleic acid (DNA) using cervical, vulva and vaginal swabs and a less invasive, probably imaging technique such as ultrasound or hysteroscopy to detect upper genital tract FGS lesions would have been useful to complement the visual lower genital tract FGS diagnoses to give more power to the findings of this study. More so, the convenience sampling method employed in this study might have introduce some selection bias causing challenges in generalizing the findings of this study to the population of women in the region.

Despite all these, the findings of this study have shed more light on the FGS burden in the Volta region of Ghana and call for more detailed and robust studies to find more on the contribution of FGS on infertility in the country.

## Conclusion

More than half of the overall participating women had visual FGS (58.5%) and most women with FGS were, fertile (76%), had childhood contact with rivers and streams (68.4%), had no toilet facilities in their homes (74.6%), practiced open defecation (77.2%), had vaginal discharge (70.3%) and complained of at least one genital symptom (65.4%).

The findings in this study call for more research on this topic to elucidate the true contribution of FGS on infertility and other clinical complications including cervical cancer and AIDS, in the region and Ghana as a whole. This can be achieved through the incorporation of complementary diagnostic techniques such as imaging, molecular profiling and other less invasive techniques such as hysteroscopy to view the upper genital tract, using more rigorous study methods such as, case–control or longitudinal study design. Additionally, FGS surveillance in the future, can be integrated into cervical cancer screening in Schistosoma-endemic communities in Ghana during planning/implementation.

## Data Availability

The dataset used and/or analyzed during the current study is available from the first author on reasonable request.

## Abbreviations

FGS: Female genital schistosomiasis
HIV: Human immunodeficiency virus
WHO: World Health Organization
GSS: Ghana Statistical Service
UHAS: University of Health and Allied Sciences
REC: Review Ethics Committee
STI: Sexually transmitted infections
HPV: Human papilloma virus
AIDS: Acquired immune deficiency syndrome
DNA: Deoxyribonucleic acid
